# Developing a highly efficient hydroxytyrosol whole-cell catalyst by de-bottlenecking rate-limiting steps

**DOI:** 10.1038/s41467-020-14918-5

**Published:** 2020-03-23

**Authors:** Jun Yao, Yang He, Nannan Su, Sakshibeedu R. Bharath, Yong Tao, Jian-Ming Jin, Wei Chen, Haiwei Song, Shuang-Yan Tang

**Affiliations:** 1 CAS Key Laboratory of Microbial Physiological and Metabolic Engineering, State Key Laboratory of Microbial Resources, Institute of Microbiology, Chinese Academy of Sciences, Beijing, China; 2 University of Chinese Academy of Sciences, Beijing, China; 3 Institute of Molecular and Cell Biology, 61 Biopolis Drive, Singapore, Singapore; 4 Beijing Key Laboratory of Plant Resources Research and Development, Beijing Technology and Business University, Beijing, China

**Keywords:** High-throughput screening, Sensors and probes, X-ray crystallography, Metabolic engineering

## Abstract

Hydroxytyrosol is an antioxidant free radical scavenger that is biosynthesized from tyrosine. In metabolic engineering efforts, the use of the mouse tyrosine hydroxylase limits its production. Here, we design an efficient whole-cell catalyst of hydroxytyrosol in *Escherichia coli* by de-bottlenecking two rate-limiting enzymatic steps. First, we replace the mouse tyrosine hydroxylase by an engineered two-component flavin-dependent monooxygenase HpaBC of *E. coli* through structure-guided modeling and directed evolution. Next, we elucidate the structure of the *Corynebacterium glutamicum* VanR regulatory protein complexed with its inducer vanillic acid. By switching its induction specificity from vanillic acid to hydroxytyrosol, VanR is engineered into a hydroxytyrosol biosensor. Then, with this biosensor, we use in vivo-directed evolution to optimize the activity of tyramine oxidase (TYO), the second rate-limiting enzyme in hydroxytyrosol biosynthesis. The final strain reaches a 95% conversion rate of tyrosine. This study demonstrates the effectiveness of sequentially de-bottlenecking rate-limiting steps for whole-cell catalyst development.

## Abstract (web summary)

Whole-cell catalyst-based hydroxytyrosol production is low. Here, the authors increase the efficiency of its production in *E. coli* by de-bottlenecking two enzymatic steps catalyzed by monooxygenase and tyramine oxidase using structure-based enzyme redesign or in vivo-directed evolution with the aid of a newly developed biosensor.

## Introduction

Whole-cell catalysts have been frequently used for natural product biosynthesis owing to their stability, recycleability and environmental friendliness. Whole-cell catalysts harboring multiple-step biosynthetic pathways avoid the time-consuming catalyst preparation process in in vitro catalysis^1^. For combinatorial whole-cell biocatalysts, the biosynthetic pathway efficiency is the most important factor influencing catalyst productivity. Besides the metabolic engineering strategies used for directing the carbon flux into a targeted pathway, the pathway enzymes are crucial for driving the pathway rates, selectivity, and overall productivity^2,3^. High-efficient pathway enzymes, especially those catalyzing rate-limiting steps, are critical for the end product productivity^4^. Rate-limiting steps of the biosynthetic pathways in combinatorial whole-cell catalysts might not be the same as those in the original hosts, due to the different protein expression levels, enzymatic activities and substrate or cofactor levels in the engineered host cells. When one rate-limiting step is unblocked, most probably another rate-limiting step will appear. A high-efficient whole-cell catalyst can be developed when all rate-limiting steps are sequentially solved.

Besides the conventional method of overexpressing enzymes catalyzing the rate-limiting steps, protein engineering is a frequently used strategy. The activity, specificity, selectivity, and allosteric property of the pathway enzymes have been engineered to improve pathway performance^4–6^. An alternative solution is to redesign the pathways to bypass rate-limiting steps or allow shortcuts for synthesis. To facilitate pathway optimization in whole-cell catalysts, the end-product biosensors are powerful tools, allowing rapid selection of hyper-producing cells^7^. The strategy of in vivo-directed evolution combined with biosensor screening is more advantageous than in vitro engineering by better adapting to the intracellular environment, including the concentration of substrates, intermediates, and cofactors, enabling the selection of hyper-producing whole-cell catalysts under real in vivo environments^8–10^. Development of customized biosensors for targeted products is challenging.

Hydroxytyrosol is a powerful antioxidant scavenger of free radicals that confers cell protection^11,12^. The hydroxytyrosol biosynthetic pathway, which uses tyrosine as a substrate, was first reported by Satoh^13^. In this pathway, the tyrosine hydroxylation step catalyzed by mouse tyrosine hydroxylase using the cofactor tetrahydromonapterin (MH4) severely limits biosynthetic efficiency, resulting in <20% hydroxytyrosol yield. Chung et al.^14^ used the aromatic aldehyde synthase AAS and the 4-hydroxyphenylacetic acid 3-hydroxylase HpaBC to turn tyrosine to hydroxytyrosol in *Escherichia coli*, however the titer of hydroxytyrosol is only 208 mg L^−1^. Li et al.^15^ established an artificial pathway for hydroxytyrosol biosynthesis from tyrosine by using the ketoacid decarboxylase KDC, the alcohol dehydrogenase ADH and also the 4-hydroxyphenylacetic acid 3-hydroxylase HpaBC, and finally obtained a hydroxytyrosol titer of ~1243 mg L^−1^ in fed-batch experiment.

In this study, we aim to demonstrate the feasibility of developing a powerful hydroxytyrosol whole-cell catalyst using *E. coli*, by sequentially solving the activities of enzymes catalyzing the rate-limiting steps of the pathway. Strategies of structure-based enzyme redesign and in vivo-directed evolution are adopted, until all the rate-limiting steps are solved and the conversion rate of end product reaches 95%.

## Results

### Development of a high-active microbial tyrosine hydroxylase

To improve the low efficiency of the tyrosine hydroxylation step catalyzed by mouse tyrosine hydroxylase^13^, a microbial tyrosine hydroxylase was redesigned by engineering HpaBC, a two-component flavin-dependent monooxygenase from *E. coli*. HpaBC catalyzes the first step in the 4-hydroxyphenylacetate (4-HPA) degradation pathway, producing 3,4-dihydroxyphenylacetate (HPC). In spite of a broad substrate spectrum, HpaBC exhibits extremely low activity on tyrosine (approximately 5% of its activity on 4-HPA)^16^.

**Fig. 1:**
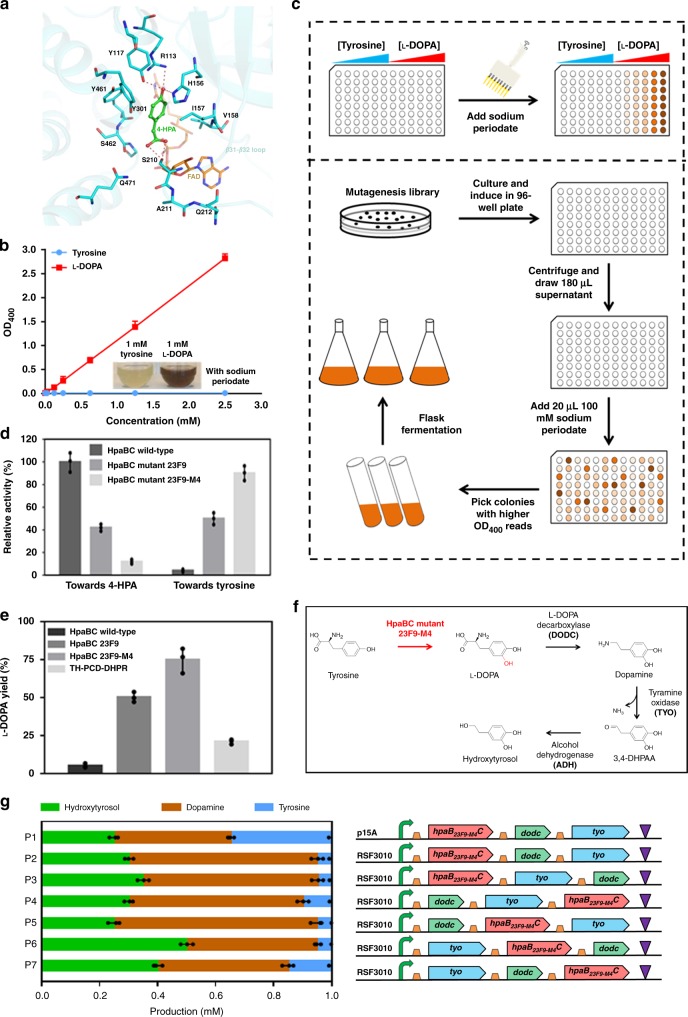
Development of a microbial tyrosine hydroxylase and redesign of the hydroxytyrosol biosynthetic pathway. **a** Model of HpaBC bound with 4-HPA and FAD. 4-HPA and FAD are shown as stick and colored in green and brown, respectively. Hydrogen bonds are shown as red dash. **b** The standard curve of the periodate assay. **c** Flowchart of the high-throughput screening of HpaBC. **d** Specific activities of the wild-type and mutant HpaBCs on 4-HPA and tyrosine. **e** l-DOPA yields of strain BW25113 expressing wild-type, HpaBC mutant 23F9, HpaBC mutant 23F9-M4 and the reported pathway enzymes TH-PCD-DHPR^13^. **f** Redesign of the hydroxytyrosol biosynthetic pathway using HpaBC mutant 23F9-M4 as a highly efficient tyrosine hydroxylase. **g** Optimization of plasmid copy number and gene orders in the operon encoding the biosynthetic pathway. The concentration of the indicated compounds was determined, and no l-DOPA was accumulated (The green, brown and blue bars indicate the concentrations of hydroxytyrosol, dopamine and tyrosine, respectively). 4-HPA, 4-hydroxyphenylacetate; 3,4-DHPAA, 3,4-dihydroxyphenyl acetaldehyde. The data shown in **b**, **d**, **e** and **g** are from three replicate experiments and are expressed as the mean±SD. Source data underlying Figs. 1b, d, e, and g are provided as a Source Data file.

To explore the mechanism of the catalytic versatility of HpaBC, we modeled an active HpaBC bound with FAD and 4-HPA based on the reported structures of EcHpaBC (*E. coli*)^17^ and ThHpaBC (*Thermus thermophiles*)^18^. We hypothesized that the binding of 4-HPA to HpaBC, like its homolog in ThHpaBC, would induce a conformational change in loop β32-33 (Supplementary Fig. 1). As shown in Fig. 1a, the hydroxyl group of 4-HPA would form hydrogen bonds with the side chains of R113, Y117, and H155, which are highly conserved and involved in the catalytic reaction. The carboxyl group of 4-HPA is predicted to be coordinated by the side chain of S210 through two hydrogen bonds. The flexibility of loop β32-33 is likely to define the substrate selectivity of HpaBC. Therefore, we chose residues S210, A211 and Q212 for saturation mutagenesis in order to evolve HpaBC into a highly active tyrosine hydroxylase which can efficiently convert tyrosine into l-DOPA.

A high-throughput screening method was developed according to the hydroxylase activity of HpaBC^19^. As shown in Fig. 1b, when reacted with sodium periodate, l-DOPA turned brown, whereas tyrosine exhibited no color change. OD_400_ can be used to quantify this colorimetric difference, and a positive linear correlation was observed between OD_400_ and l-DOPA concentrations. Therefore, the sodium periodate assay was used for high-throughput screening of the l-DOPA formation activity of HpaBC in 96-well plates (Fig. 1c). The saturation mutagenesis library was transformed into strain BW25113 and subjected to high-throughput screening. From ~50,000 clones, 12 mutant strains that exhibited improved tyrosine hydroxylation capability compared with that of the strain expressing the wild-type enzyme were selected (Supplementary Fig. 2). The mutant HpaBC expressed by mutant strain exhibiting the highest hydroxylation activity, 23F9, was purified for activity assay, along with the wild-type enzyme. As shown in Fig. 1d, the relative hydroxylase activity of the 23F9 mutant on tyrosine was ~12-fold higher than that of the wild-type enzyme. To further improve the tyrosine hydroxylation activity, a random mutagenesis library was constructed based on mutant 23F9. High-throughput screening of this library resulted in six mutant strains showing increased absorbance at 400 nm (Supplementary Fig. 3a) and produced high titers of l-DOPA (Supplementary Fig. 3b), compared with those of strain expressing the 23F9 mutant. The HpaBC mutant 23F9-M4 exhibited a 1.8-fold higher hydroxylation activity on tyrosine than that of the 23F9 mutant (Fig. 1d). Furthermore, the hydroxylation activity of these HpaBC mutants on the original substrate 4-HPA reduced considerably (Fig. 1d). The plasmids expressing these HpaBC mutants were purified from the selected mutant strains and used to retransform strain BW25113, and enhanced l-DOPA yields was confirmed. The yield of l-DOPA from strains expressing mutant 23F9 and 23F9-M4 was found to increase by ~10- and 15-fold, respectively, compared with that of the strain expressing wild-type HpaBC (Fig. 1e). The l-DOPA yield from strain expressing the mouse tyrosine hydroxylase and the MH4 regeneration cycle constructed according to Satoh et al.^13^ was ~21%, around 4-fold of that of the strain expressing wild-type HpaBC (Fig. 1e). The sequencing results revealed amino acid substitutions in the selected HpaB mutants (Table 1). Therefore, a highly active microbial tyrosine hydroxylase was successfully redesigned to replace mouse tyrosine hydroxylase.

**Table 1:** Amino acid substitutions in variants.

Protein	Amino acid substitutions				
HpaBC	15	210	211	212	284
Wild-type	T	S	A	Q	D
23F9	T	F	K	F	D
23F9-M4	P	F	K	F	E
VanR	103	148	168		
Wild-type	Y	R	Y		
HyT5	K	I	L		
HyT12	L	L	M		
HyT19	N	V	P		
HyT31	P	V	N		
HyT55	G	R	P		
HyT134	I	S	P		
HyT172	T	M	W		
TYO	33	60	103	149	238
Wild-type	E	H	D	D	V
YM9	E	Q	Y	E	V
YM9-2	D	Q	Y	E	E

### Redesign of the hydroxytyrosol bi3osynthetic pathway

The HpaBC mutant 23F9-M4 was used to redesign the hydroxytyrosol biosynthetic pathway, with the removal of the regeneration system of cofactor MH4^13^ (Fig. 1f). The redesigned pathway was constructed via a plasmid expressing an operon consisting of genes encoding mutant 23F9-M4, l-DOPA decarboxylase (DODC), and tyramine oxidase (TYO), as well as the *E. coli* endogenous alcohol dehydrogenase (ADH). As the knockout of gene *feaB* has been reported to be essential to achieve high yield and high purity of hydroxytyrosol^13^, we knocked out the *feaB* gene in strain BW25113 to obtain strain BHYT^19^. The plasmid copy number and the order of the three genes in the operon were optimized, in order to improve the hydroxytyrosol biosynthesis efficiency (Fig. 1g). Plasmid P2 carrying the RSF3010 replication origin resulted in high hydroxytyrosol production compared with that of the plasmid carrying the p15A replication origin. The gene order in plasmid P6 presented the highest hydroxytyrosol production (Fig. 1g). It was found that with the redesigned tyrosine hydroxylation step with plasmid P6, the tyrosine conversion rate reached 95%, which indicated that it was no longer a rate-limiting step. Although this rate-limiting step was solved, the hydroxytyrosol yield of the whole pathway was only around 50%. By analyzing the intracellular concentrations of biosynthetic intermediates, it was found that dopamine accumulated in large amounts in strain BHYT harboring plasmid P6 (Fig. 1g), indicating that the oxidation of dopamine to 3,4-dihydroxyphenyl acetaldehyde (3,4-DHPAA) was insufficient. This reaction was catalyzed by TYO, and it has become the rate-limiting step of the redesigned pathway. As this reaction generated NH_3_ and H_2_O_2_ which are toxic to the cell in high amounts, to avoid toxic intermediate accumulation, balanced activities between TYO and downstream pathway enzymes are critical for efficient hydroxytyrosol biosynthesis. Therefore, in vivo-directed evolution was performed to optimize the TYO activity. To minimize the negative effect of the toxic intermediates on biosynthesis, a high-throughput screening tool of the end product would help. Thus, a hydroxytyrosol biosensor was developed by engineering the regulatory protein VanR.

### Crystal structure of VanR with bound vanillic acid

**Fig. 2:**
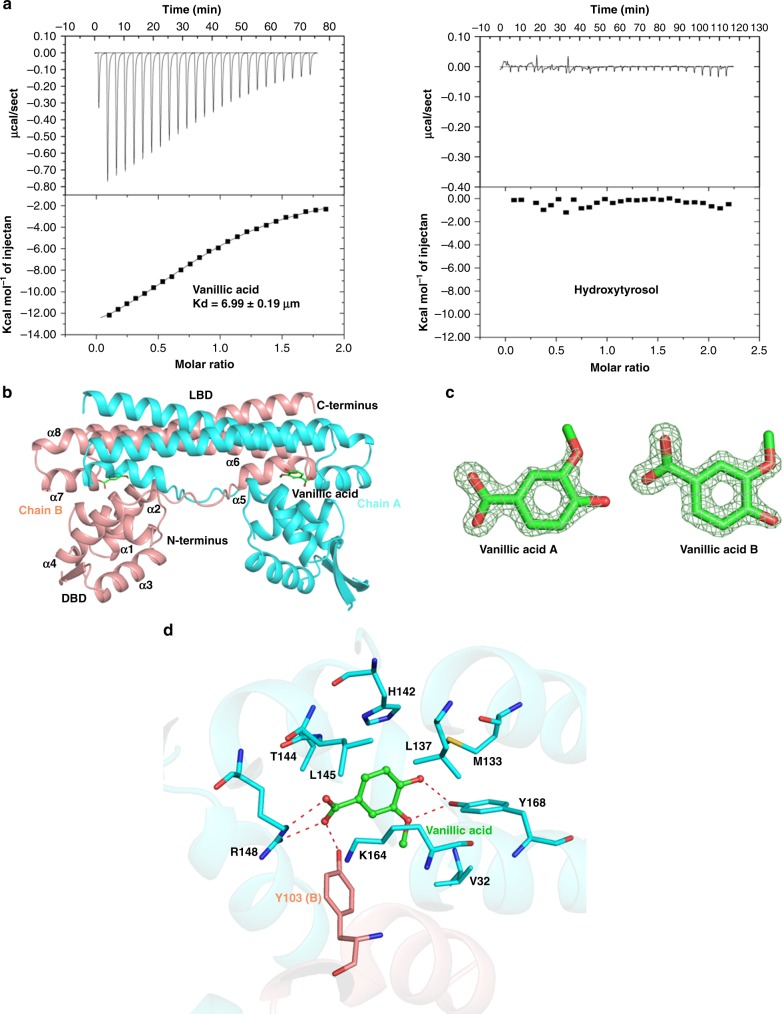
Crystal structure elucidation of VanR complexed with vanillic acid. **a** ITC titrations of vanillic acid and hydroxytyrosol to wild-type VanR. The upper panels show the binding isotherms and the lower panels show the integrated heat for each injection fitted to a single-site model. **b** Crystal structure of the VanR with bound vanillic acid. Subunits A and B are colored in cyan and light red respectively. **c** Fo-Fc electron density map of vanillic acid at the contour of 2.0 colored in light green; **d** The interaction of VanR with vanillic acid. Vanillic acid is shown in green stick and polar contacts are shown as red dash lines.

A regulatory protein from *C. glutamicum*, VanR, was chosen for effector specificity engineering to develop a hydroxytyrosol-responsive regulator. VanR belongs to the PadR family of regulatory proteins and has been proved to specifically respond to its effetor vanillic acid, a hydroxytyrosol analog^20^. As shown in Fig. 2a, vanillic acid binds to VanR with moderate affinity (*K*_d_ = 6.99 μM), but no binding was observed between hydroxytyrosol and VanR.

To facilitate VanR engineering, we determined the crystal structure of VanR complexed with vanillic acid (Supplementary Table 1). VanR functions as a homodimer and each monomer consists of an N-terminal winged helix-turn-helix (wHTH) domain which interacts with operator DNA, and a C-terminal homodimerization domain (Fig. 2b). Electronic densities of vanillic acids were found in the interface of two subunits (Fig. 2c, Supplementary Fig. 4a, b, Supplementary Fig. 5). Vanillic acid binds to a hydrophobic interdomain pocket formed by V29, Y168, M133, L145, and H142 from subunit A and Y103 of subunit B (Fig. 2d). Besides hydrophobic interaction, polar contacts largely contribute to the recognition of vanillic acid by VanR. Specifically, the carboxyl group of vanillic acid is electrostatically stabilized by salt bridges with R148 of subunit A and by hydrogen bond with the side chain of Y103 of subunit B. Furthermore, the hydroxyl and methoxyl groups on the benzene ring of vanillic acid form two hydrogen bonds with the side chains from Y168 of subunit A (Fig. 2d). Those three residues were highly conserved in different bacteria (Supplementary Fig. 4c), suggesting they are essential for ligand recognition.

### Development of a hydroxytyrosol-responsive VanR mutant

**Fig. 3:**
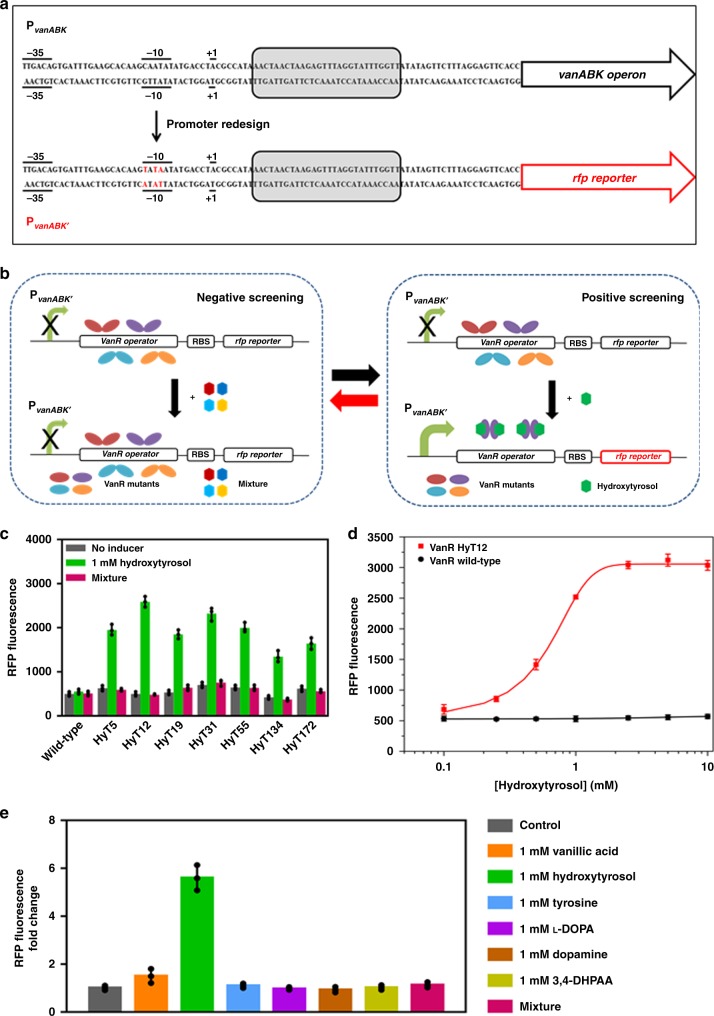
Adaptation of the VanR regulatory system in *E. coli* and its induction specificity alteration. **a** Adaptation of the VanR regulatory system in *E. coli* by redesigning the −10 region of P_*vanABK*_. The substituted base pairs are highlighted in red. **b** High-throughput screening strategy for engineering the induction specificity of VanR. **c** RFP fluorescence of strain BW25113 harboring plasmid pVanR expressing wild-type or mutant VanRs in the absence of any inducer (black bar) or in the presence of 1 mM hydroxytyrosol (green bar) or in the presence of the mixture (Magenta bar). **d** RFP fluorescence from strain BW25113 harboring plasmid pVanR expressing VanR wild-type (black circle) or the HyT12 mutant (red square) as a function of the hydroxytyrosol concentrations. **e** RFP fluorescence of strain BW25113 harboring plasmid pVanR expressing the HyT12 mutant in response to the indicated compounds. Mixture represents a mixture of 1 mM each of tyrosine, l-DOPA, dopamine and 3,4-DHPAA. The data shown in **c**, **d** and **e** are from three replicate experiments and are expressed as the mean±SD. Source data underlying Fig. 3c-e are provided as a Source Data file.

In *C. glutamicum*, VanR regulates the expression of the *vanABK* operon, which is responsible for the catabolism of vanillic acid. In the absence of vanillic acid, the VanR protein binds to a 28-bp operator sequence downstream of +1 position of the P_*vanABK*_ promoter and represses the transcription of the *vanABK* operon. When vanillic acid is present, it binds to the VanR protein, inducing a conformational change which releases the promoter DNA, thus activating the operon transcription^20^. Directly introducing the whole promoter region (95-bp) into *E. coli* cells could not initiate downstream gene transcription (data not shown). To adapt a functional VanR regulatory system in *E. coli*, after sequence analysis, the −10 region of promoter P_*vanABK*_ (CAATAT) was replaced with the −10 region of the *tac* promoter (P_*tac*_) (TATAAT) frequently used in *E. coli* (Fig. 3a) to generate promoter P_*vanABK*_’. The resultant promotor successfully regulated the downstream *rfp* gene expression in response to vanillic acid in the presence of the constitutively expressed VanR protein in *E. coli*, with plasmid pVanR (Supplementary Figs. 6, 7).

To alter the effector specificity of VanR, amino acid residues Y103, R148, and Y168, were chosen for simultaneous site-saturation mutagenesis (Fig. 2d). The library was constructed using plasmid pVanR and used to transforming strain *E. coli* BW25113 (3.5 × 10^5^ transformants). The transformants were screened by fluorescence-activated cell sorting (FACS). One round of negative screening was performed by sorting the least fluorescent cells (2.8 × 10^5^) in the presence of 1 mM each of tyrosine, l-DOPA, dopamine and 3,4-DHPAA, in order to remove the VanR variants responsive to the substrate and intermediates in hydroxytyrosol biosynthesis. One round of positive screening was then performed by sorting the most fluorescent cells (3.5 × 10^3^) in the presence of 1 mM hydroxytyrosol (Fig. 3b). After seven rounds of dual screening, seven variants responsive to hydroxytyrosol but not the substrate and intermediates were selected. Among them variant HyT12 exhibited the highest induction fold towards hydroxytyrosol and was used for downstream characterizations (Fig. 3c). The sequencing results revealed amino acid substitutions (Y103L, R148L and Y168M) in HyT12. Figure 3d depicts RFP fluorescence of strain BW25113 expressing variant HyT12, as a function of hydroxytyrosol concentration. Approximately 6-fold induction of RFP expression was achieved over a hydroxytyrosol concentration range of 0–10 mM, with half-maximal induction occurring at ~0.5 mM. Wild-type VanR exhibited no response towards hydroxytyrosol. The induction effects of the biosynthetic substrate tyrosine and the intermediates l-DOPA, 3,4-DHPAA and dopamine, as well as the original inducer vanillic acid, were determined individually, by comparing with those of hydroxytyrosol. As shown in Fig. 3e, the HyT12 variant showed a good specific response towards hydroxytyrosol, compared with that of the other tested compounds, indicating that negative screening using a mixture of the biosynthetic substrate and intermediates was definitely effective. This induction specificity is critical for the application of HyT12 variant as a hydroxytyrosol biosensor for high-throughput screening. Additionally, we modeled the binding of hydroxytyrosol to the VanR mutants. As shown in the modeling data in Supplementary Fig. 8a, we found a significantly higher Rosseta function score of HyT12 than that of the other mutants, indicating a considerable conformational change in HyT12 to accommodate the ligand. As depicted in Supplementary Fig. 8b, hydroxytyrosol would form hydrogen bond with the side chain of T144 and H142, and the hydroxyl tail was accommodated by a hydrophobic cavity formed by V32, K164, A167, M168 from subunit A and A102 and L103 from subunit B. The amino acid substitutions in various VanR variants are shown in Table 1.

### Application of a whole-cell hydroxytyrosol biosensor

**Fig. 4:**
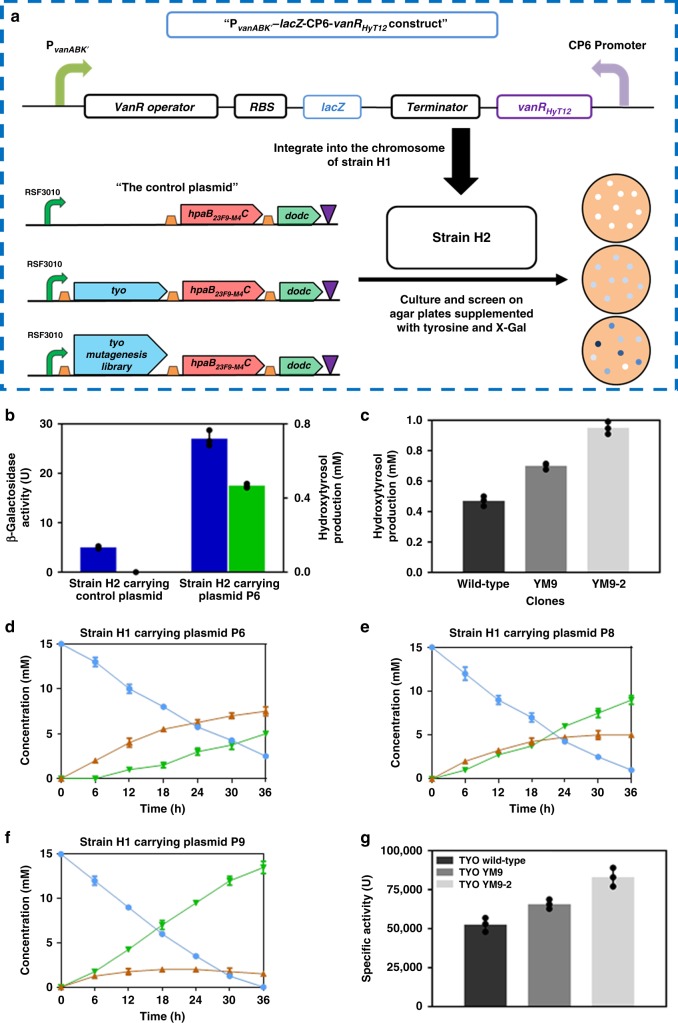
Design of a hydroxytyrosol biosensor and its application in TYO activity optimization. **a** Design of a hydroxytyrosol biosensor and the strategy of applying it in in vivo-directed evolution of TYO for improved hydroxytyrosol biosynthesis. **b** β-Galactosidase activity (blue) and hydroxytyrosol productions (green) in strain H2 harboring control plasmid and plasmid P6, after culturing at 37 °C for 36 h. **c** Hydroxytyrosol productions in the wild-type and selected clones in the first and second rounds of in vivo-directed evolution, after culturing at 37 °C for 36 h, in the presence of 1 mM tyrosine; Time courses of productions of hydroxytyrosol and dopamine, as well as tyrosine consumption, from strain BHYT carrying plasmid P6 (**d**), P8 (**e**), or P9 (**f**) in a 5-L fermentor. Blue circle, brown triangle and green triangle indicate the concentration of tyrosine, dopamine and hydroxytyrosol, respectively. **g** Specific activities of wild-type and mutant TYOs. The data shown in **b**, **c**, **d**, **e**, **f** and **g** are from three replicate experiments and are expressed as the mean±SD. Source data underlying Fig. 4b-g are provided as a Source Data file.

As depicted in Fig. 4a, strain H2 was constructed by integrating the constitutively expressed HyT12 and the P_*vanABK*_’-*lacZ* construct into the chromosome of strain BHYT. We used β-galactosidase encoded by *lacZ* as a reporter to facilitate the subsequent screening on agar plates. Strain H2 was then transformed with plasmid P6 or the control plasmid without the *tyo* gene absent. As shown in Fig. 4b, the results indicated that the developed whole-cell hydroxytyrosol biosensor could effectively report intracellular hydroxytyrosol production.

In vivo-directed evolution was used to optimize the activity of TYO in the biosynthetic pathway and the hydroxytyrosol whole-cell biosensor was used for high-throughput screening. A random mutagenesis library of TYO was constructed with plasmid P6 and was used to transform strain H2. The deep blue colonies, which were supposed to be hydroxytyrosol hyper-producers, were selected on agar plates supplemented with 3 mM tyrosine and 40 µg mL^−1^ 5-bromo-4-chloro-3-indolyl β-d-galactopyranoside (X-GAL). Twelve clones were selected from 1.0 × 10^6^ transformants screened, and they were re-screened by quantifying hydroxytyrosol production from liquid culture. Variant YM9 was found to produce the highest amount of hydroxytyrosol (Supplementary Fig. 9a). To further optimize the TYO activity, a random mutagenesis library was constructed using variant YM9 as a template, and the library was subjected to high-throughput screening as described above except with 6 mM tyrosine supplementation. Six clones exhibiting high hydroxytyrosol production were selected, and variant YM9-2 produced the highest amount of hydroxytyrosol (Supplementary Fig. 9b). As shown in Fig. 4c, when cultured in the presence of 1 mM tyrosine, clones YM9 and YM9-2 displayed a ~1.49- and 2.02-fold increase in hydroxytyrosol production, respectively, compared with that of the strain harboring pathway expressing wild-type TYO. The hydroxytyrosol yield of clone YM9-2 reached 95%. Sequencing results revealed amino acid substitutions in the mutated TYOs (Table 1).

As shown in Fig. 4d-f, the time course of the concentration of hydroxytyrosol and intermediates, as well as the consumption of tyrosine, was determined, with strain BHYT harboring plasmids P6, P8 (plasmid P6 expressing TYO YM9), and P9 (plasmid P6 expressing TYO YM9-2) cultured in a 5-L fermentor. The strains were cultured in the presence of 15 mM tyrsosine. For strain harboring plasmid P6, the hydroxytyrosol yield was 50% and a large amount of dopamine was accumulated. In strain harboring plasmid P8 (pathway expressing the TYO mutant YM9), dopamine accumulation was alleviated and hydroxytyrosol production was improved. As for strain harboring plasmid P9 (pathway expressing the TYO mutant YM9-2), the accumulation of dopamine was considerably reduced and the hydroxytyrosol yield reached 90% (Fig. 4f), comparing with 33.3% in the presence of wild-type TYO (Fig. 4d).

Fed-batch fermentation and whole-cell biotransformation were both performed to produce hydroxytyrosol from tyrosine. In fed-batch fermentation, strain harboring plasmid P9 was cultured in a 5-L fermentor in the presence of 1 mM l-arabinose, and 7.5 mM of tyrosine was fed with at 0, 12, 24 and 36 h. The time course of hydroxytyrosol production was analyzed (Supplementary Fig. 10). It was found that after 48 h at 37 °C, the hydroxytyrosol production was ~3,373 mg L^−1^. In whole-cell biotransformation, cells harboring plasmid P9 was resuspended in 1 L (OD_600_ = 50) of 100 mM Tris-HCl buffer (pH 7.5) with 15 mM tyrosine. An additional 30 mM of tyrosine was supplemented at 12 h and the biotransformation was extended to 48 h at 37 °C. A rotating speed of 100 rpm and air flow rate of 1.0 vvm were used, with pH maintained at 7.5. The final hydroxytyrosol production reached 4,690 mg L^−1^, which was higher than reported so far.

### Analysis and characterization of wild-type and mutant TYOs

The genes encoding wild-type or mutant TYOs were cloned into the pET28a vector and expressed in *E. coli* BL21(DE3) as N-terminal His-tagged fusion proteins. The enzymes were then purified for activity assay and kinetic parameter determination. As shown in Fig. 4g, the TYO variants YM9 and YM9-2 exhibited ~1.3- and ~1.6-fold increase in specific activity, respectively, relative to that of the wild-type enzyme. This corresponded well with hydroxytyrosol productions (Fig. 4c**)**. The results indicated that the improved activity of the rate-limiting enzyme, TYO, underlies enhanced hydroxytyrosol productions in the selected hyper-producing strains. The kinetic parameters of the mutant TYOs were determined, along with the wild-type enzyme (Table 2). Our data revealed that the affinity of the mutant enzymes for substrate dopamine increased. The catalytic efficiency (*k*_cat_/K_m_) of the mutant enzymes YM9 and YM9-2 showed ~1.7- and ~3.3-fold improvements compared with that of the wild-type enzyme, respectively (Table 2).

**Table 2:** Kinetic parameters of wild-type and mutant TYOs.

Enzymes	K_m_	*k*_cat_	*k*_cat_/K_m_
TYO wild-type	8.48 ± 1.06	115.11 ± 21.95	13.52 ± 1.22
TYO YM9	6.67 ± 0.72	156.71 ± 16.99	23.48 ± 0.27
TYO YM9-2	4.30 ± 0.37	194.42 ± 16.43	45.20 ± 0.45

± values represent standard deviations from three independent data points. Source data are provided as a Source Data file.

## Discussion

Tyrosine hydroxylase is the key enzyme in the biosynthesis of catecholamines dopamine, epinephrine and norepinephrine in animals, which are hormones and neurotransmitters in both the central and peripheral nervous systems^21^. Tyrosine hydroxylase uses tetrahydrobiopterin as a cofactor, and its activity is regulated by phosphorylation and also feedback inhibited by the catecholamine neurotransmitters^22^. Due to its complicated regulation properties and the necessity for cofactor regeneration cycle, acting as the first catalytic step, it severely reduces the biosynthesis efficiency of the hydroxytyrosol pathway^13^. To redesign this pathway, a non-natual microbial tyrosine hydroxylase was developed by engineering substrate specificity of the two-component flavin-dependent monooxygenase, HpaBC, from *E. coli* strain BL21(DE3). The wild-type HpaBC showed negligible activity on tyrosine^16^. By engineering its substrate-binding pocket, a highly active tyrosine hydroxylase was obtained, which possessed no allosteric inhibitory property and was expressed very well in *E. coli* cells. Thus, it was suitable for replacing the animal-sourced tyrosine hydroxylase for hydroxytyrosol biosynthesis in *E. coli*. The conversion rate of tyrosine significantly increased in the presence of this microbial tyrosine hydroxylase.

Innate transcriptional regulatory protein (TRP)-based biosensors are widely-used in natural product high-throughput screening^23–26^. However, under most circumstances, innate transcription factors responsive to targeted compounds are not available. Studies on development and application of non-natural transcription factors with altered specificity are limited^8–10^. Since there is no regulatory protein responsive to hydroxytyrosol reported so far, a non-natural hydroxytyrosol biosensor has been customized by altering the induction specificity of VanR regulatory protein. The specificity of a biosensor used as a high-throughput screening tool to optimize the pathway efficiency is critical, because it should not respond to the biosynthetic substrate or intermediates. However, performing negative screenings for each of substrate and intermediates in hydroxytyrosol biosynthetic pathway is time-consuming. Thus, a mixture of these compounds was used in a single round of negative screening, while hydroxytyrosol was used in the positive screening. A VanR mutant specifically responsive to hydroxytyrosol was successfully selected, indicating the effectiveness of this screening strategy. Thus the strategy for customizing biosensors for small molecules synthesized through multiple-step pathways has been advanced.

Structural information provides key clues for redesigning proteins including enzymes, sensors, and probes. Polar contacts between ligand and protein, including hydrogen bond and salt bridge, are very critical for redesigning. The crystal structures of both HpaBC and VanR are essential for the development of this redesigned synthetic pathway of hydroxytyrosol. Binding of substrate to HpaBC induced a conformational transition of loop from an open state to a closed state. The flexibility of this loop probably allows different analogs to enter the catalytic pocket. Changes in amino acids of this loop probably result in a different selectivity of substrates. In our model, S210 had a polar contact with 4-HPA, however, A211 and Q212 could also be involved in the recognition of ligands due to flexibility. T196 and A197 of ThHpaBC, located at similar positions as S210 and A211 of EcHpaBC would form hydrogen bond with 4-HPA. As for VanR, all three residues had polar contacts with vanillic acid and were chosen to engineer in our screening. Although HyT12 showed the highest response to the ligand, the other six variants also demonstrate comparable effects. Interestingly, we found the highest Rosetta function score in HyT12 that showed the highest response to ligand. This indicates that changes in the overall conformation of VanR might be important for its function, as the highest response of HyT12 might come from the release of DNA in the DBD domain upon ligand binding. Compared to rational design, the semi-rational design might be more efficient in our case.

Balancing pathway enzyme activities has been proved critical for pathway efficiency^23,24^. The reaction catalyzed by TYO, the rate-limiting enzyme of the redesigned hydroxytyrosol pathway, generated ammonia and hydrogen peroxide^25,26^, thus, an optimal activity instead of extremely high activity would benefit the whole pathway efficiency. In vivo-directed evolution combined with high-throughput screening of the end product is a good strategy for optimizing this enzyme to achieve improved hydroxytyrosol biosynthetic efficiency, by avoiding the toxicity caused by intermediate accumulation. In vivo-directed evolution is advantageous for pathway engineering because it better optimizes the metabolic fluxes by adapting to the intracellular environment, which could not be well managed by in vitro protein engineering strategies. On the contrary, for the rate-limiting enzymes with extremely low activity, it is more rapidly to improve the pathway efficiency by simply replacing them with redesigned enzymes, for example, the redesigned microbial tyrosine hydroxylase described above.

Engineering pathway rate-limiting enzyme to improve biosynthetic efficiency has been adopted in combinatorial biosynthesis. However, unblocking one rate-limiting step does not necessarily solve the overall efficiency of the whole-cell catalysts, especially when the catalytic steps are more than three. Subsequent rate-limiting steps usually come into being. Sequentially identifying and solving these steps until the overall conversion rate reaches close to 100% is the final goal and crucial task of whole-cell catalyst development. The strategies for de-bottlenecking rate-limiting steps can be diverse, depending on the features of the steps. Apart from the two strategies used in this study, other strategies include cofactor regeneration, branch pathway blocking, and so on.

As a value-added compound with a variety of biological activities, hydroxytyrosol biosynthesis has been reported through various pathways^13–15,19^. Previously, with tyrosine as substrate, hydroxytyrosol productions of 29.29 mg L^−1^ (~19% yield)^13^, 208 mg L^−1^ ^14^, 1243 mg L^−1^ (~48% yield)^15^ and 1890 mg L^−1^ (82% yield)^19^ have been achieved. l-DOPA is used for treating Parkinson’s disease and also a precursor for synthesizing valued benzylisoquinoline alkaloids^27,28^. Various biotechnological approaches have been applied for l-DOPA production, including microbial fermentation, immobilized tyrosinase as well as electroenzymatic system^29^. Previous studies have revealed an over 3.5 mg L^−1^ of l-DOPA production from glucose in yeast, using a tyrosine hydroxylase developed from a cytochrome P450 l-DOPA oxidase^30,31^. In our study, hydroxytyrosol production from fed-batch fermentation has reached ~3373 mg L^−1^. Whole-cell transformation further improved the production to ~4690 mg L^−1^. Furthermore, the HpaBC mutant 23F9-M4, which synthesized 443.7 mg L^−1^ l-DOPA from 3 mM tyrosine, is also potentially applicable for l-DOPA production.

In conclusion, in this study, the first rate-limiting enzyme in the hydroxytyrosol biosynthetic pathway, mouse tyrosine hydroxylase, was replaced with an artificially designed high-efficient microbial tyrosine hydroxylase. This redesigned step converted 95% of tyrosine to l-DOPA, and the rate-limiting step of the pathway moved to dopamine oxidation catalyzed by TYO. The second rate-limiting step was optimized by in vivo-directed evolution, with the aid of a hydroxytyrosol biosensor. The crystal structure of the regulatory protein VanR from *C. glutamicum* complexed with its inducer was elucidated based on which a hydroxytyrosol biosensor was developed by altering the induction specificity of VanR. With this biosensor as an end product high-throughput screening tool, the TYO activity was optimized, further improving the hydroxytyrosol yield to 95%, the highest as reported to date^13–15,19^. This study has demonstrated that a powerful whole-cell biocatalyst can be developed by sequentially de-bottlenecking the rate-limiting steps, which should be generally applied in other combinatorial biosynthesis studies.

## Methods

### General

The biosynthetic substrate, product and intermediates used in this study were obtained from Sigma-Aldrich (St. Louis, USA). The T4 polynucleotide kinase, T4 DNA ligase, restriction enzymes and DNA polymerase used in this study were obtained from Takara Bio (Dalian, China). Hieff Clone Plus Multi One Step Cloning Kit was obtained from YEASEN Biotechnology (Shanghai, China). Oligonucleotides were synthesized by Life Technologies (Shanghai, China).

*E. coli* strains MC1061 and BL21(DE3) were used for cloning and protein overexpression, respectively. Strains were grown at 37 °C in the indicated medium. Ampicillin (100 μg mL^−1^) and kanamycin (50 μg mL^−1^) were supplemented when required. Hydroxytyrosol biosynthesis was performed in yeast extract M9Y medium (M9 minimal salts, 1% (w/v) glucose, 5 mM MgSO_4_, 0.1 mM CaCl_2_ supplemented with 0.025% (w/v) of yeast extract)^13^, while other cultures were done in Luria-Bertani (LB) medium if not specially indicated.

### Plasmid construction

All plasmids and primers used in this study are listed in Supplementary Data 1 and 2, respectively. The *dodc* gene encoding l-DOPA decarboxylase (Genbank accession No. BK006920.1) was amplified from the genome of strain *Pseudomonas putida* KT2440. The *tyo* gene from *Micrococcus luteus* encoding tyramine oxidase (Genbank accession No. AB010716.1) was synthesized after codon optimization^19^. The purified gene fragments *hpaB*_*23F9-M4*_*C*, *dodc*, *tyo, tyo*_*MUT1*_*, tyo*_*MUT1-22*_ were assembled with the vector fragment of pFA or pRSF^19^, using Hieff Clone Plus Multi One Step Cloning Kit (YEASEN, Shanghai, China), resulting in plasmids pFA-*hpaB*_*23F9-M4*_*C-dodc*-*tyo* (P1), pRSF- *hpaB*_*23F9-M4*_*C-dodc*-*tyo* (P2), pRSF- *hpaB*_*23F9-M4*_*C*-*tyo*-*dodc* (P3), pRSF-*dodc*-*tyo*- *hpaB*_*23F9-M4*_*C* (P4), pRSF-*dodc*- *hpaB*_*23F9-M4*_*C-tyo* (P5), pRSF-*tyo*- *hpaB*_*23F9-M4*_*C*-*dodc* (P6), and pRSF-*tyo-dodc- hpaB*_*23F9-M4*_*C* (P7), pRSF-*tyo*_*MUT1*_- *hpaB*_*23F9-M4*_*C*-*dodc* (P8), pRSF-*tyo*_*MUT1-22*_- *hpaB*_*23F9-M4*_*C*-*dodc* (P9). The genes were cloned in different orders in the operons.

The *th* gene encoding tyrosine hydroxylase (TH) from mouse (Genbank accession No. NP_033403), the *pcd* gene encoding pterin-4α-carbinolamine dehydratase (PCD) from human (Genbank accession No. NP_000272) and the *dhpr* gene encoding dihydropteridine reductase (DHPR) from human (Genbank accession NO.P09417) were synthesized by General Biosystems (Chuzhou,China) after codon optimization (Supplementary Data 3). The DNA fragments containing genes *th*, *pcd, dhpr* and vector pRSF^19^ were amplified using primer pairs *th*-Gibson-1/*th*-Gibson-2, *pcd*-Gibson-1/*pcd*-Gibson-2, *dhpr*-Gibson-1/*dhpr*-Gibson-2 and pRSF-Gibson-1/pRSF-Gibson-2, respectively, and then were assembled using Hieff Clone Plus Multi One Step Cloning Kit (YEASEN, Shanghai, China), resulting in plasmid pRSF-*th-pcd*-*dhpr*.

The fragment containing promoter region of *vanABK* operon (P_*vanABK*_) was amplified using the genome of strain *C. glutamicum* ATCC 13032 as template with primers P_*vanABK*_-Gibson-1 and P_*vanABK*_-Gibson-2. The vector fragment was amplified using plasmid pSHYa as template with primers pSHYa-*backbone*-Gibson-1 and pSHYa-*backbone*-Gibson-2. The two purified fragments were assembled using Hieff Clone Plus Multi one Step Cloning Kit (YEASEN, Shanghai, China), resulting in plasmid pVan. The mutations in the promoter region were introduced with Stratagene QuikChange Site-Directed Mutagenesis Kit (Agilent Technologies Inc, Santa Clara, USA).

The fragment containing the *vanR* gene encoding transcriptional regulatory protein (Genbank accession No. 1020332) was amplified using the genome of strain *C. glutamicum* ATCC 13032 as template with primers *vanR*-Gibson-1 and *vanR*-Gibson-2. Using plasmid pVan as template, amplification was performed with primers pVanR-*backbone*-Gibson-1 and pVanR-*backbone*-Gibson-2. The two purified fragments were assembled using Hieff Clone Plus Multi One Step Cloning Kit, resulting in plasmid pVanR.

The wild-type or mutant *vanR* genes were amplified using plasmid pVanR as template with primers *VanR*-for-*EcoR*I and *VanR*-rev-*Xho*I, and ligated into plasmid pGEX-6p-1 after digestion with *EcoR*I and *Xho*I, resulting in plasmid pGEX-6p-1-*vanR* carrying wild-type or mutant *vanR* gene.

### Strain construction and culture conditions

The *lacZ* gene was amplified from the genomic DNA of strain MG1655 with primers *lacZ*-for-*Kpn*I and *lacZ*-rev-*Bgl*II and ligated into plasmid pVanR carrying gene encoding the VanR mutant HyT12, after digestion with *Kpn*I and *Bgl*II. Then the fragment containing P_*vanABK’*_ controlled *lacZ* (P_*vanABK’*_-*lacZ*) and gene encoding the VanR mutant HyT12 was amplified with primers pAH156-P_*vanABK’*_-for and pAH156-P_*vanABK’*_-rev, and assembled with the PCR fragment amplified with primers *vanR*-pAH156-for and pAH156-P_*vanABK’*_-rev using the CRIM plasmid pAH156^32^ as template, by Gibson Assembly. The construct was then integrated into the chromosome of strain BHYT using helper plasmid pAH69^32^, resulting in strain H2. The integration was verified by PCR.

### Library construction

A site-saturation mutagenesis library of HpaBC with amino acid residues S210, A211 and Q212 in the HpaB component randomized was constructed on plasmid pFA-*hpaBC*^19^ with primers *hpaBC*-Saturated-for and *hpaBC*-Saturated-rev^19^. Around 10^5^ transformants were collected for downstream screening.

To construct a random mutagenesis library based on the HpaB mutant 23F9, Error-prone PCR was performed using plasmid pFA-*hpaB*_*23F9*_*C* as template with primers *hpaB*-ErrorProne-for and *hpaB*-ErrorProne-rev. The PCR reaction was done as described^19^. The PCR products obtained, containing randomly mutated *hpaB*_*23F9*_ gene, were used as the megaprimer to perform the MEGAWHOP PCR^33^ using plasmid pFA-*hpaB*_*23F9*_*C* as template. *Dpn*I (20 U) digestion was performed at 37 °C for 2 h and then inactivated at 80 °C for 20 min. Then the PCR products were used to transform strain *E. coli* MC1061 and around 10^5^ transformants were recovered. Randomly picked clones from the libraries were sequenced, and an average of 2.5 nucleotide mutations per clone was confirmed. All colonies from the agar plates were used for plasmid isolation to prepare the plasmid library.

To construct the site-saturation mutagenesis library of VanR, two parallel PCR reactions were performed with plasmid pVanR as template using the following two sets of primers: *vanR1*-for/*vanR1*-rev and *vanR2*-for/*vanR2*-rev. The two amplified fragments, *vanR*-F1 and *vanR*-F2, contained the site-saturation mutagenesis at positions 103, 143 and 168. Equimolar aliquots of the two fragments (1.6 nmole each) were PCR-assembled without primers, and then primers *vanR*-F0-for and *vanR*-F0-rev were added to amplify the PCR products. The PCR products were used as megaprimer to perform MEGAWHOP PCR using pVanR as template as described above. Around 5×10^4^ transformants were recovered. Randomly picked clones from the library were sequenced, and the mutations at the indicated positions were confirmed with no additional point mutations.

The construction of the random mutagenesis library of TYO was similar to the HpaBC random mutagenesis library as described above, except that primer pairs *tyo*-ErrorProne-for/*tyo*-ErrorProne-rev and template plasmid pRSF-*tyo-hpaB*_*23F9-M4*_*C-dodc* carrying the gene encoding TYO wild-type or mutant MUT1 were used. A total of 10^4^ transformants were recovered and randomly picked clones were sequenced and contained an average of 3 (library of wild-type *tyo*) or 2.5 (library of *tyo-mut1*) nucleotide mutations per clone.

### High-throughput screening of the mutagenesis libraries

The HpaBC library was screened with the periodate assay method^34^. Single colonies of strain BHYT harboring mutants from the site-saturation mutagenesis library of HpaB or random mutagenesis library of HpaB mutant 23F9 were grown in 0.6 mL M9Y medium in 96-well plates at 37 °C for 24 h, induced with 1 mM l-arabinose and supplemented with 3 mM tyrosine as substrate. Cells were harvested by centrifugation (1840 × *g*, 10 min, 4 °C). 180 μL of the supernatant was added into 20 μL of 100 mM sodium periodate, and OD_400_ of the reaction mixture was determined by a SynergyMx Multi-Mode Microplate Reader (BioTek, Vermont, USA). Mutants showing higher absorbance than the parent enzymes were selected.

The VanR plasmid library was used to transform strain BW25113 (3.5 × 10^5^ transformants were recovered). FACS screening was performed on a FACSAria II sorter (BD, San Jose, USA). Fluorescence was excited at 561 nm and the fluorescence emission was detected using a 610/20 nm band-pass filter. In the first round of negative screening, cells were precultured overnight in LB medium and then diluted to OD_600_ = 0.2 in the same medium in the presence of 1 mM tyrosine, 1 mM l-DOPA, 1 mM dopamine and 1 mM 3,4-DHPAA. The cells were then grown for 12 h and the least fluorescent 2.8 × 10^5^ cells were collected from a total of 3.5 × 10^5^ cells, to eliminate clones that were induced by substrate and biosynthetic intermediate compounds. The collected cells were then induced with 1 mM hydroxytyrosol and grown for 12 h to enable positive screening, in which the most fluorescent 3.5 × 10^3^ cells were sorted from a total of 3.5 × 10^5^ cells (Supplementary Fig. 11). These itinerary screens were repeated for seven times, and seven clones were selected for rescreening in test tubes.

The TYO random mutagenesis libraries were screening on agar plates. The libraries were used to transform strain H2. After incubation at 37 °C for 24 h on YM9 agar supplemented with 3–6 mM tyrosine, 1 mM l-arabinose, 20 μg mL^−1^ X-GAL and 50 μg mL^−1^ kanamycin, the darkest blue colonies (by the eye) were selected for quantification of hydroxytyrosol productions in liquid culture.

### HPLC quantification

A colony of strain BW25113 harboring plasmid pFA-*hpaBC*^19^ carrying wild-type or mutant *hpaBC* gene, or strain BHYT harboring the hydroxytyrosol biosynthetic pathway, was grown in M9Y medium at 37 °C till OD_600_ = 0.6, then the cells were induced with 1 mM l-arabinose and 3 mM tyrosine was supplemented. After further grown at 37 °C for 24 h, the cell culture was centrifuged at 12,000 × g for 5 min, and the supernatant was filtrated through a 0.22 μm filter and analyzed with HPLC. Concentrations of l-DOPA, tyrosine and hydroxytyrosol were determined by HPLC^19^. The concentrations of dopamine were determined after derivation with dansylcholoride, detected at 254 nm, by HPLC^19^.

### Protein expression and purification

A single colony of strain BL21(DE3) harboring plasmid pGEX-6p-1-*vanR* carrying wild-type or mutant *vanR* (Y103L, R148L and Y168M), was grown in LB medium at 37 °C till OD_600_ = 0.6, then induced with 0.4 mM IPTG. Full length of wild-type and mutant VanR were expressed as GST fusion protein in strain BL21(DE3). Proteins were purified using a glutathione Sephararose 4B column. After the GST-tag was cleaved by PreScission protease (GE Healthcare), the proteins were further purified by a second glutathione Sephararose 4B column, the collected fractions containing target proteins were then loaded into MonoQ column for further purification with a linear gradient elution, and fractions containing VanR or mutant VanR from MonoQ were finally purified through Supperdex 75 (GE Healthcare) column equilibrated with buffer (20 mM Tris, pH 8.0, 200 mM NaCl, 2 mM DTT). The wild-type and mutant VanR were concentrated to 10 mg mL^−1^ and stored at −80 °C for use.

The purification of HpaB and HpaC were performed using nickel-nitrilotriacetic acid (Ni-NTA) column (Qiagen, Valencia, USA)^19^. Sodium dodecyl sulfate polyacrylamide gel electrophoresis (SDS-PAGE) was used to assess the protein purity. Protein concentration was assayed with Bradford method^35^.

### Crystallization and structure determination

Purified VanR was concentrated to 7 mg mL^−1^. VanR and vanillic acid were incubated for on ice 2 h before crystallization. Crystal was obtained in 0.2 M magnesium chloride, 0.1 M Tris pH 8.0, 12% PEG4000 by hanging drop method at 18 °C. Then the crystal was flash-cooled in liquid nitrogen to −170 °C. The diffraction data were collected at 100 K at the synchrotron radiation at beam line of Shanghai Synchrotron Radiation Facility. The diffraction data sets were integrated and scaled with XDS package^36,37^. The structure of VanR complexed with vanillic acid was solved by molecular replacement using the structure of smu.1604c from *Streptococcus* mutants UA159 (https://www.rcsb.org/structure/3L9F) as the initial model with Phaser^38^. Refmac 5 and Coot9 in CCP4 suite were used for the refinement and model building^39,40^.

### Isothermal titration calorimetry

For isothermal titration calorimetry (ITC measurement), the purified wild-type or mutant VanR was dialysis overnight at 4 °C against 20 mM phosphate buffer containing 150 mM sodium chloride, pH 7.4. Vanillic acid was dissolved in the same buffer. Isothermal titration calorimetry was carried out at 25 °C on an ITC200 system (MicroCal). 200 μM vanillic acid was titrated into 20 μM VanR. A titration of vanillic acid into buffer was carried out as a control. Data were fitted and analyzed using Origin 7 (MicroCal).

### Molecular modeling

To generate the active HpaBC complexes with FAD and 4-HPA, we modeled an active structure using ThHpaBC (https://www.rcsb.org/structure/2YYJ) as a template by Modoller 9.20^18,41^. We replace the loop of apo HpaBC (https://www.rcsb.org/structure/6QW0) with the previously modeled loop (residues 206–218) by YASARA. Then the model was further minimized using GROMACS under force field gromacs 96^42^. Finally, FAD and 4-HPA were docked to model by Autodock Vina^43^.

The structures of screened VanR mutants were manually generated in Coot^44^. Then Rosseta ligand docking^45,46^ was used to calculate the binding model. The conformation with lowest docking score were choose as final model, and Rosseta functions were used to evaluate the change of mutant proteins.

### Activity assay of HpaBC and TYO

The activity assay of TYO was performed at 37 °C by monitoring the absorbance increase at 240 nm which indicates the formation of H_2_O_2_^47^. A reaction mixture (200 μL) for the assay contained 100 mM phosphate buffer (pH 7.0), 0.75 mM dopamine and 0.002 mg mL^−1^ purified TYO protein. Reaction was performed at 37 °C for 10 min. The H_2_O_2_ formation was monitored at 240 nm, with a SynergyMx Multi-Mode Microplate Reader (BioTek, USA). One unit of activity was defined as the amount of enzyme that catalyzes the production of 1 μM of H_2_O_2_ per min.

The HpaBC activity was assayed by quantifying the product formation with HPLC. One unit of activity was defined as the amount of enzyme catalyzing the conversion of 1 mM tyrosine per minute under the assay conditions^19^.

### Kinetic parameters determination

The kinetic parameters of TYO enzyme were determined by Lineweaver-Burk plots. TYO activity assay was performed as described above at different dopamine concentrations (0.75~1.50 mM).

### Measurement of β-galactosidase activities

The β-galactosidase activity was measured using the substrate o-nitrophenyl-β-D-galactopyranoside (ONPG)^48^. Crude enzyme extracts were prepared by ultrasonication, and 10 μL of the crude enzyme extract was incubated with 140 μL of McIlvaine buffer (200 mM Na_2_HPO_4_, 100 mM citric acid, pH 6.0) containing 1 mM ONPG at 37 °C for 10 min. The reaction was stopped by adding 150 μL of 200 mM Na_2_CO_3_. The absorbance increase at 420 nm resulted from the release of o-nitrophenol (ONP) was measured. One unit (U) of enzyme activity was defined as the amount of enzyme required to liberate the equivalent of 1 μmoL of ONP perminute under the assay conditions.

### Reporting summary

Further information on research design is available in the Nature Research Reporting Summary linked to this article.

## Supplementary information

Supplementary Information

Reporting Summary

Description of Additional Supplementary Files

Supplementary Data 1

Supplementary Data 2

Supplementary Data 3

## Data Availability

A reporting summary for this article is available as a Supplementary Information file. Data supporting the findings of this work are available within the paper and its Supplementary Information files. The datasets generated and analyzed during the current study are available from the corresponding author upon request. Structure of VanR complexed with vanillic acid has been deposited in the Protein Data Bank under accession code of 6LG2 [https://www.rcsb.org/structure/6LG2]. The source data underlying Figs. 1b, d, e, g, 3c-e, and 4b-g, Table 2, as well as Supplementary Figs. 2, 3, 7, 8a, 9, and 10 are provided as a Source Data file.

## Source Data

Source Data
