# Supplementary material for: Developing a highly efficient hydroxytyrosol whole-cell catalyst by de-bottlenecking rate-limiting steps
Source: Nat Commun. 2020 Mar 23;11:1515. doi: 10.1038/s41467-020-14918-5 (PMC7090077; doi:10.1038/s41467-020-14918-5)
Supplement: Supplementary file 1 — Supplementary Information [file 41467_2020_14918_MOESM1_ESM.pdf]

Developing a highly efficient hydroxytyrosol whole-cell catalyst by de-bottlenecking rate-limiting steps

Yao *et al*.

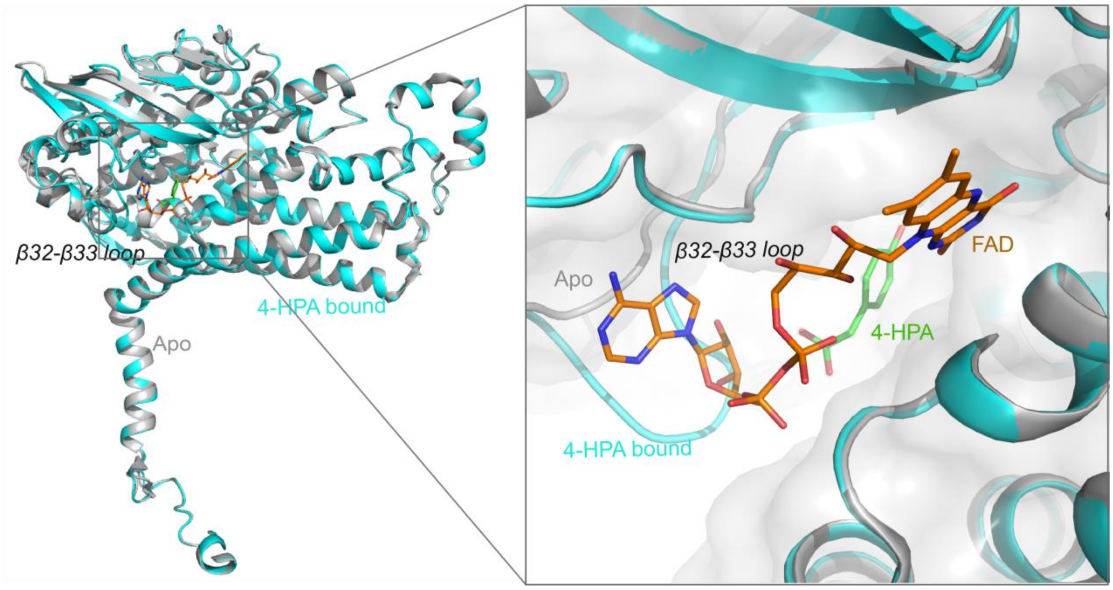

Supplementary Figure 1. Modeling of HpaBC bound with 4-HPA and FAD. The apo form (6QW0) were colored as grey, the modeled active from were colored as cyan. Docked 4-HPA and FAD were shown as green and brown stick, respectively.

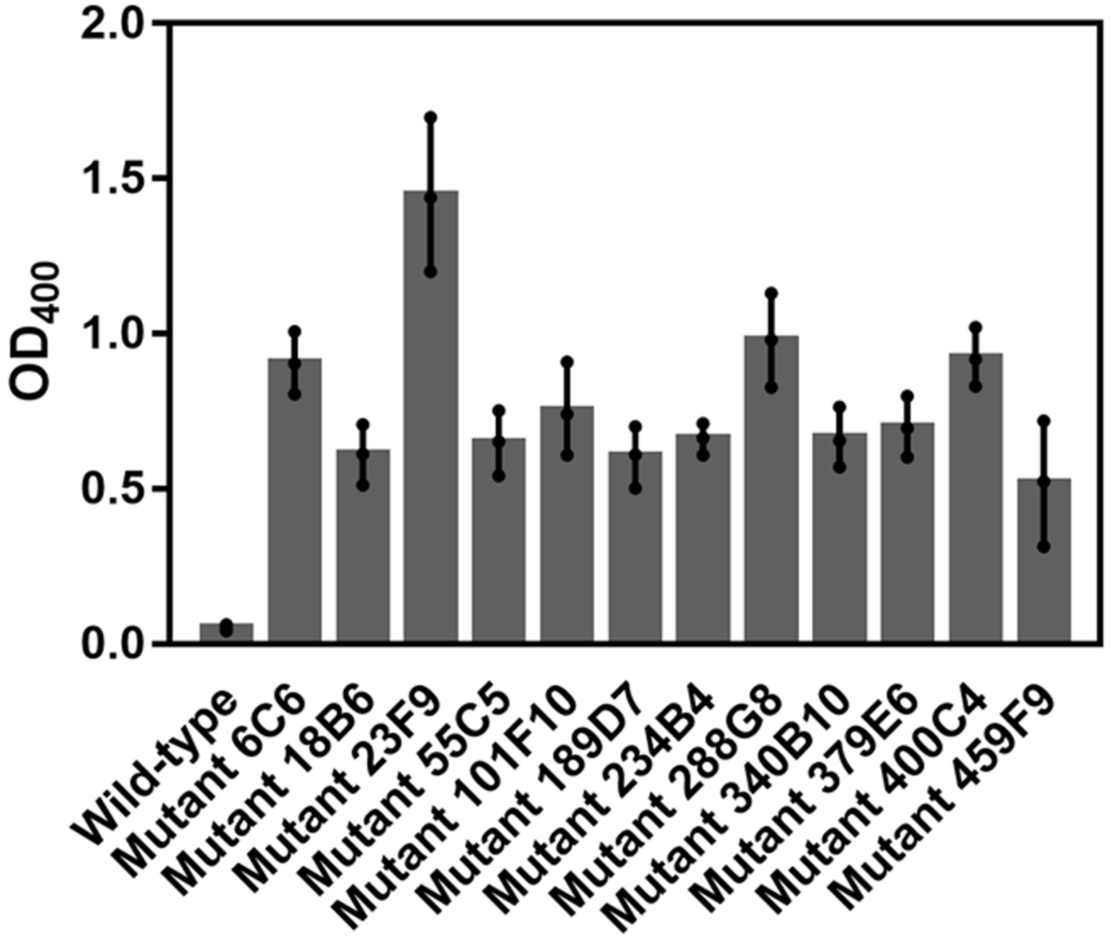

Supplementary Figure 2. OD_{400} of the periodate assay of wild-type HpaBC and 12 mutants selected in the first round of mutagenesis, using tyrosine as substrate. The data shown are from three replicate experiments and are expressed as the mean±SD. Source data are provided as a Source Data file.

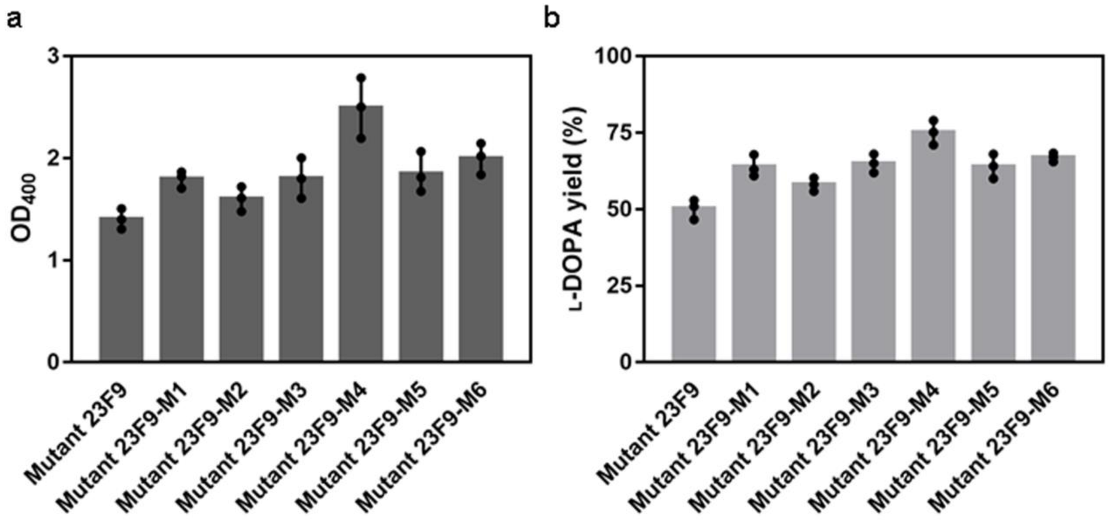

Supplementary Figure 3. Characterization of mutant HpaBCs obtained in the second round of mutagenesis. OD_{400} of the periodate assay (a) and L-DOPA yield (b) of strain 25113 expressing mutant 23F9 or the 6 mutants selected in the second round of mutagenesis. The data shown are from three replicate experiments and are expressed as the mean±SD. Source data are provided as a Source Data file.

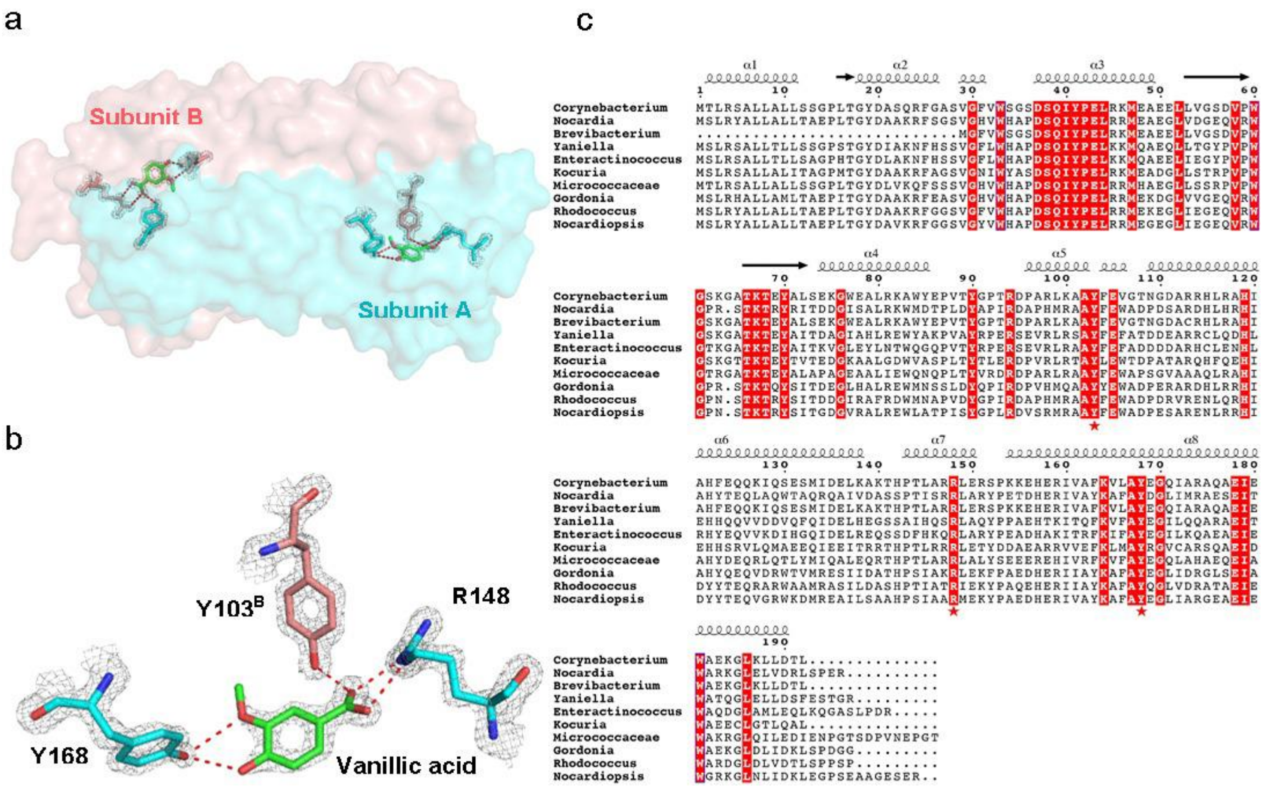

Supplementary Figure 4. Residues involved in ligand recognition are highly conserved in VanR family. (a) Vallinic acid binds to the pocket located in the interface of two subunits. Subunits A and B are shown as surfaces and colored in cyan and salmon respectively. Vallinc acids and hydrogen bonded residues are shown as sticks. (b) Fo-Fc electron densities covering vanillic acid and hydrogen bonded residues are shown in grey mesh and contoured at 2.0 s. Hydrogen bonds are shown as red dash lines. (c) Structure-based sequence alignment of VanR from different bacteria. The secondary structural elements are indicated at the top of the alignment. Consensus residues are highlighted in red, and those involved in hydrogen bond formation are marked with pentagram.

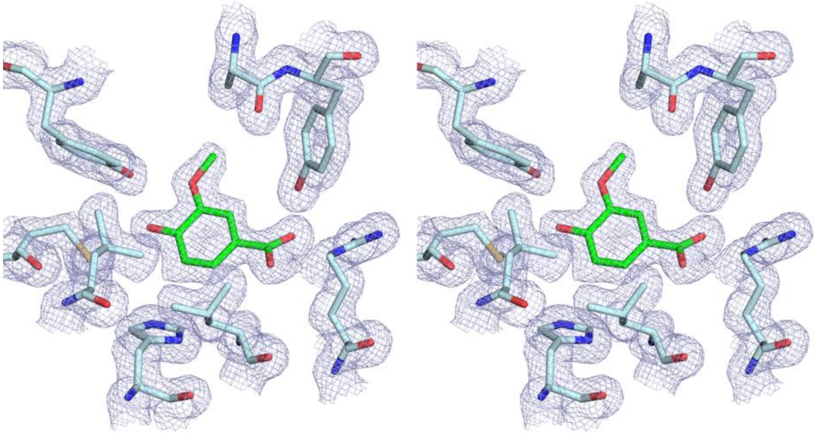

Supplementary Figure 5. Stereo image of a portion of electron density of the crystal structure of VanR complexed with vanillic acid. $2F_O - F_C$ density contoured at 1σ around vanillic acid is shown as blue mesh.

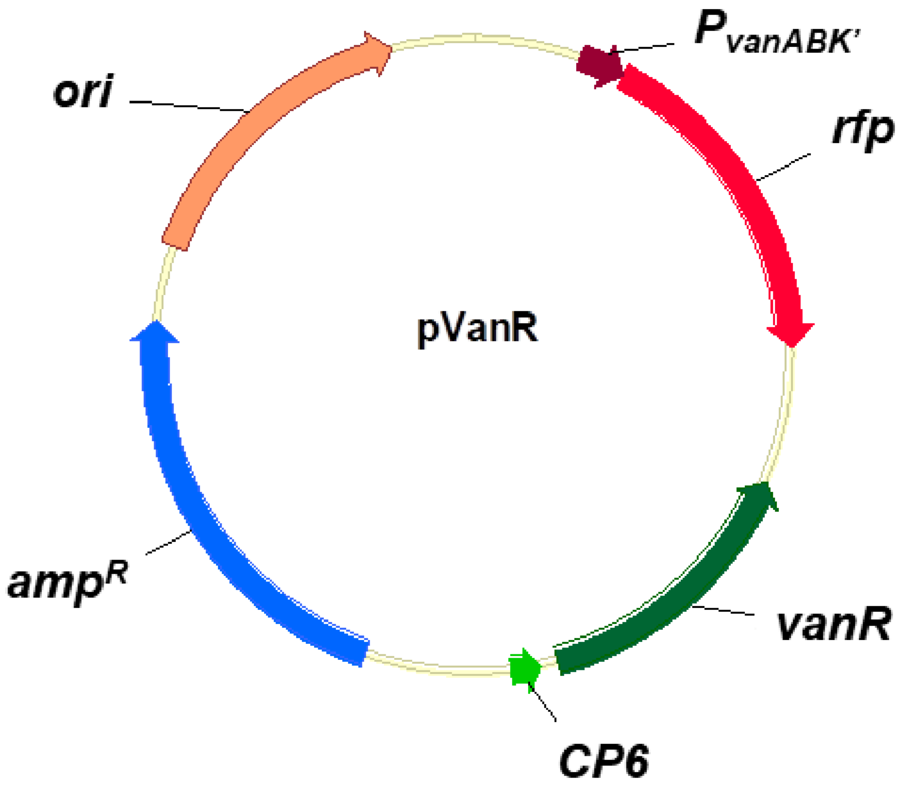

Supplementary Figure 6. Plasmid map of pVanR.

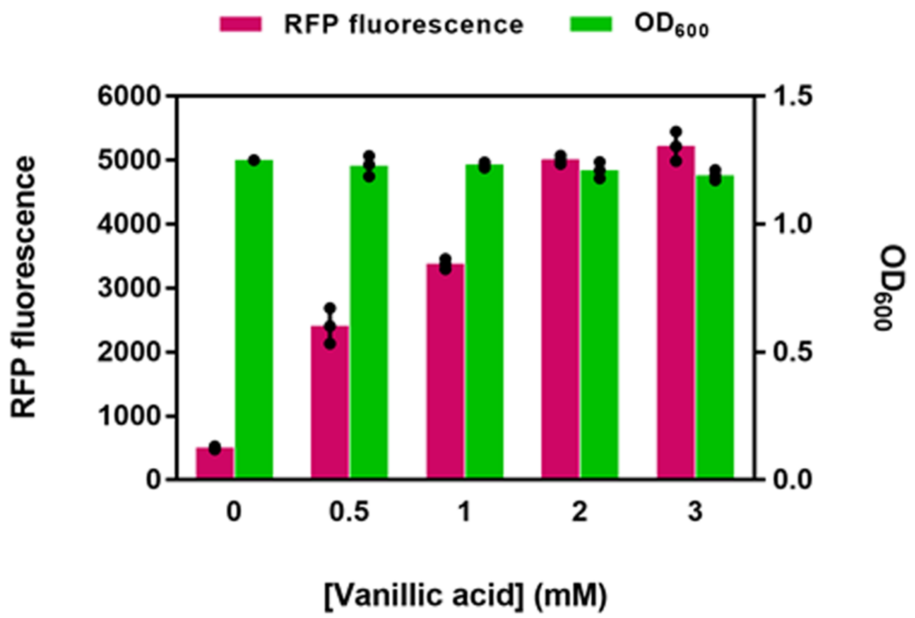

Supplementary Figure 7. Functional validation of the VanR regulatory system adapted in *E. coli*. RFP fluorescence (Magenta) and cell growth (green) of strain BW25113 carrying plasmid pVanR cultured in the presence of the indicated concentrations of vanillic acid at 37°C for 18 h. The data shown are from three replicate experiments and are expressed as the mean±SD. Source data are provided as a Source Data file.

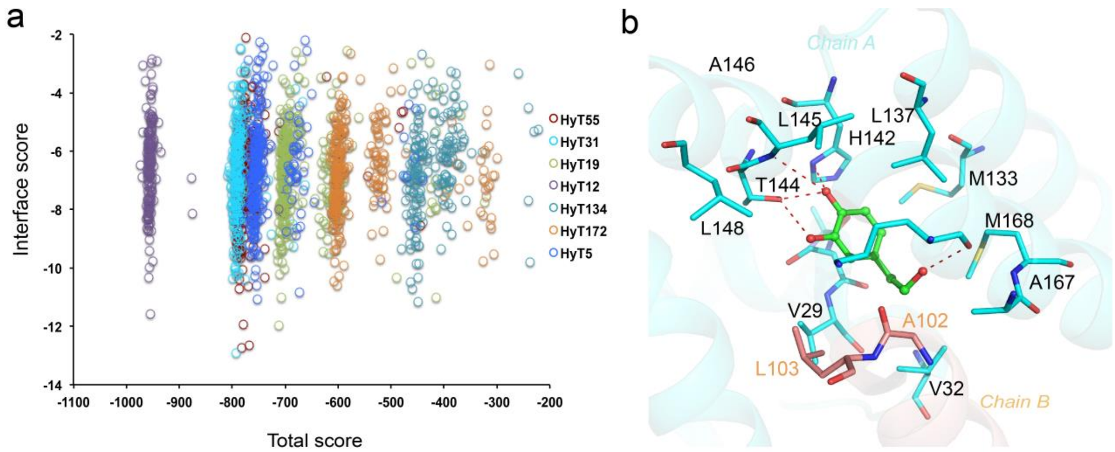

Supplementary Figure 8. Modeling of VanR mutants bound with hydroxytyrosol. (a) Docking scores and total scores of all mutants from Rosseta ligand docking. (b) Model of HyT12 bound with hydroxytyrosol, hydroxytyrosol were shown as green stick, and polar contacts were shown as red dash. Source data of Supplementary Figure 8a are provided as a Source Data file.

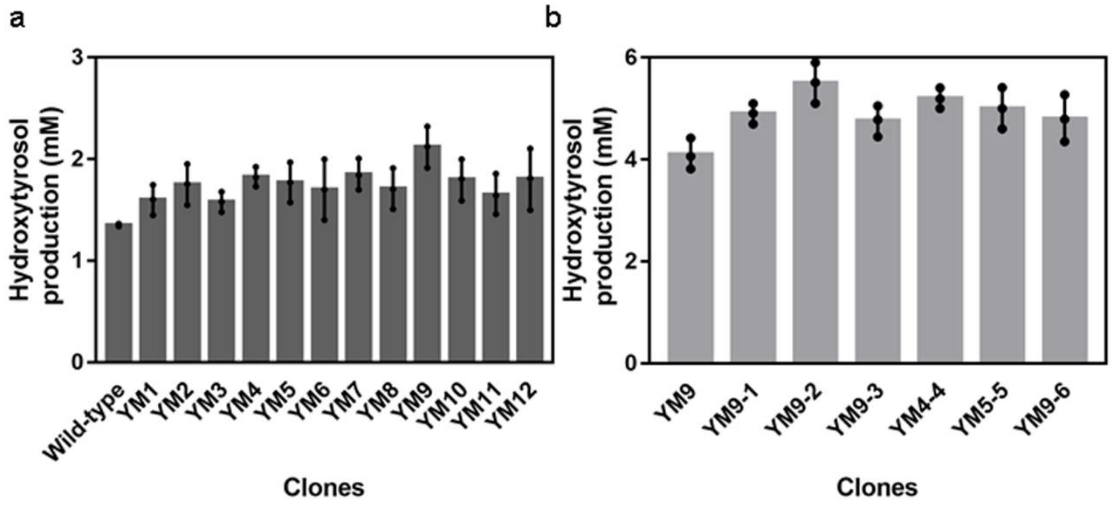

Supplementary Figure 9. ***In vivo* directed evolution of TYO.** Hydroxytyrosol productions of wild-type and selected mutant clones in the first (a) and second (b) round of mutagenesis. The data shown are from three replicate experiments and are expressed as the mean±SD. Source data are provided as a Source Data file.

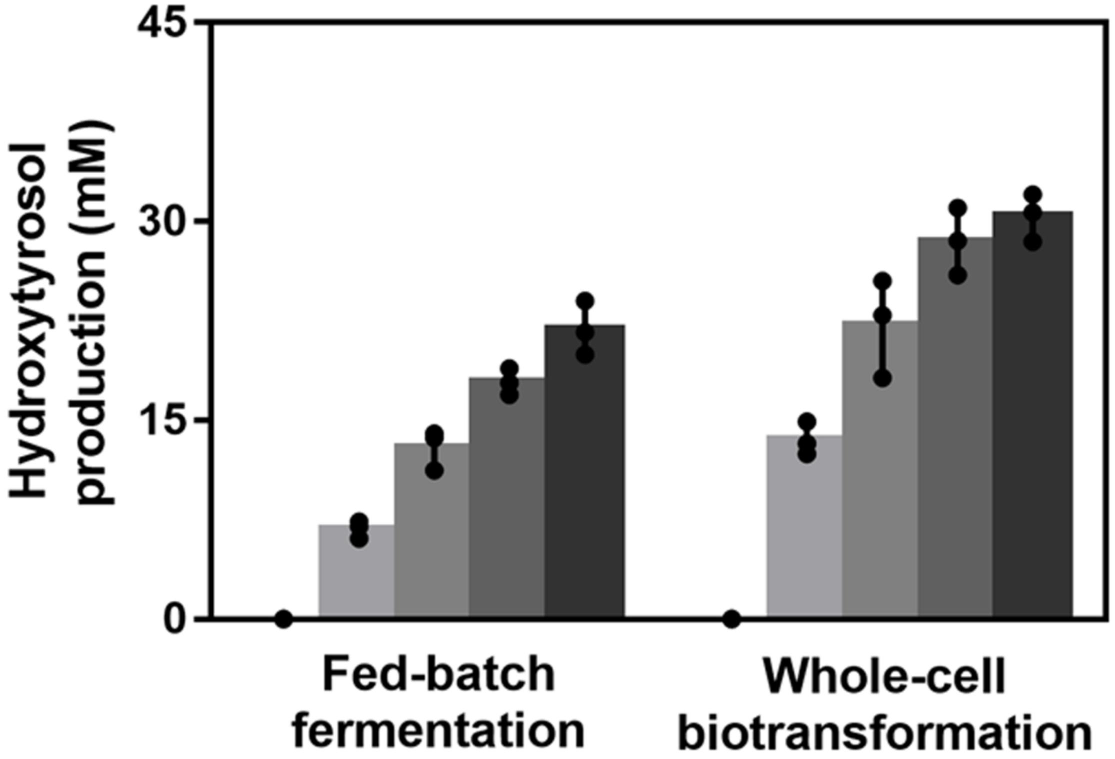

Supplementary Figure 10. Hydroxytyrosol productions from fed-batch fermentation and whole-cell transformation experiments. Strain BHYT carrying plasmid P9 was cultured in a 5-L fermentor. Columns from light gray to black represent hydroxytyrosol productions at 12, 24, 36 and 48 h of culturing. The data shown are from three replicate experiments and are expressed as the mean±SD. Source data are provided as a Source Data file.

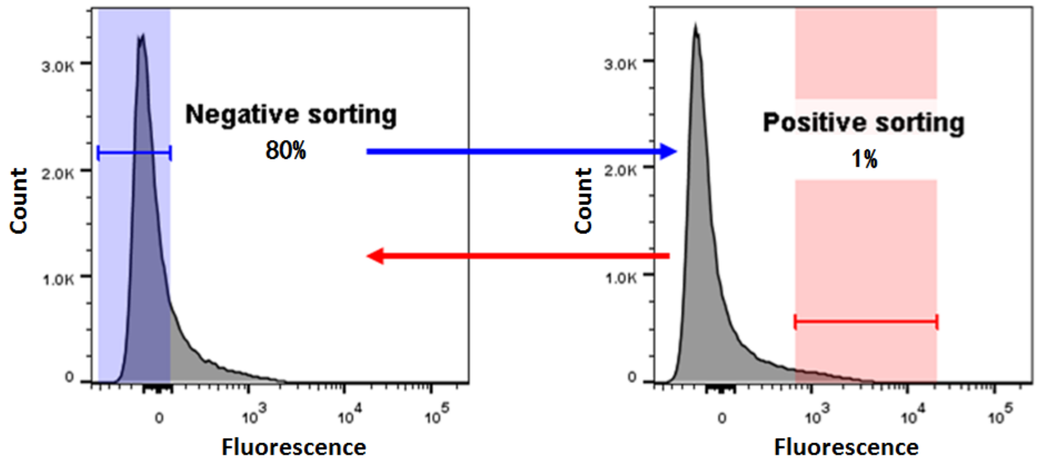

Supplementary Figure 11. FACS sorting strategy for VanR library screening. In negative screening, the least fluorescent 80% cells were sorted in the presence of 1 mM tyrosine, 1 mM L-DOPA, 1 mM dopamine and 1 mM 3,4-DHPAA; In positive screening, the most fluorescent 1% cells were sorted in the presence of 1 mM hydroxytyrosol.

Supplementary Table 1. Data collection and refinement statistics.

	VanR-vanillate
**Data collection**	
Space group	P 2_1
Cell dimensions	
a, *b*, *c* (Å)	34.97, 83.56, 63.28
α, β, γ (°)	90, 92.22, 90
Resolution (Å)	29.57-1.60 (1.63-1.60)
R_{merge}	0.097 (0.69)
$I / \sigma I$	6.3 (1.2)
Completeness (%)	93.0 (92.7)
Redundancy	1.8 (1.8)
**Refinement**	
Resolution (Å)	29.57-1.6 (1.657-1.6)
No. reflections	44437 (4436)
R_{work} / R_{free}	0.19 (0.24)
No. atoms	
Protein	2921
Ligand/ion	24
Water	335
B-factors	
Protein	17.3
Ligand/ion	17.9
Water	27.2
R.m.s. deviations	
Bond lengths (Å)	0.006
Bond angles (°)	0.772

*Number of xtals for each structure =1. Values in parentheses are for highest-resolution shell.
