# Supplementary material for: Developing a highly efficient hydroxytyrosol whole-cell catalyst by de-bottlenecking rate-limiting steps
Source: Nat Commun. 2020 Mar 23;11:1515. doi: 10.1038/s41467-020-14918-5 (PMC7090077; doi:10.1038/s41467-020-14918-5)
Supplement: Supplementary file 2 — Reporting Summary [file 41467_2020_14918_MOESM2_ESM.pdf]

natureresearch

Corresponding author(s): Shuang-Yan Tang
Last updated by author(s): Jan 13, 2020

Reporting Summary

Nature Research wishes to improve the reproducibility of the work that we publish. This form provides structure for consistency and transparency in reporting. For further information on Nature Research policies, see Authors & Referees and the Editorial Policy Checklist.

Statistics

For all statistical analyses, confirm that the following items are present in the figure legend, table legend, main text, or Methods section.

n/a	Confirmed	
☐	☒	The exact sample size (*n*) for each experimental group/condition, given as a discrete number and unit of measurement
☐	☒	A statement on whether measurements were taken from distinct samples or whether the same sample was measured repeatedly
☐	☒	The statistical test(s) used AND whether they are one- or two-sided<br>*Only common tests should be described solely by name; describe more complex techniques in the Methods section.*
☐	☒	A description of all covariates tested
☒	☐	A description of any assumptions or corrections, such as tests of normality and adjustment for multiple comparisons
☐	☒	A full description of the statistical parameters including central tendency (e.g. means) or other basic estimates (e.g. regression coefficient) AND variation (e.g. standard deviation) or associated estimates of uncertainty (e.g. confidence intervals)
☒	☐	For null hypothesis testing, the test statistic (e.g. *F*, *t*, *r*) with confidence intervals, effect sizes, degrees of freedom and *P* value noted<br>*Give P values as exact values whenever suitable.*
☒	☐	For Bayesian analysis, information on the choice of priors and Markov chain Monte Carlo settings
☒	☐	For hierarchical and complex designs, identification of the appropriate level for tests and full reporting of outcomes
☒	☐	Estimates of effect sizes (e.g. Cohen's *d*, Pearson's *r*), indicating how they were calculated

Our web collection on statistics for biologists contains articles on many of the points above.

Software and code

Policy information about availability of computer code

Data collection	BD FACSDiva™ software v6.1.3; CCP4 Suite, XDS package, GROMACS, Modoller 9.20
Data analysis	Excel 2007, Prism 7, FlowJo v7.6.1, origin 7

For manuscripts utilizing custom algorithms or software that are central to the research but not yet described in published literature, software must be made available to editors/reviewers. We strongly encourage code deposition in a community repository (e.g. GitHub). See the Nature Research guidelines for submitting code & software for further information.

Data

Policy information about availability of data

All manuscripts must include a data availability statement. This statement should provide the following information, where applicable:
- Accession codes, unique identifiers, or web links for publicly available datasets
- A list of figures that have associated raw data
- A description of any restrictions on data availability

A reporting summary for this article is available as a Supplementary Information file. The source data underlying Figure 1b, 1d, 1e, 1g, 3c, 3d, 3e, 4b, 4c, 4d, 4e, 4f, 4g, Supplementary Figures 2, 3a, 3b, 7, 8a, 9a, 9b, 10 and Table 2 can be found in the Source Data files.
The structure of VanR complexed with vanillic acid has been deposited in the Protein Data Bank [www.pdb.org], with a PDB ID code of 6LG2.

Field-specific reporting

Please select the one below that is the best fit for your research. If you are not sure, read the appropriate sections before making your selection.

☒ Life sciences ☐ Behavioural & social sciences ☐ Ecological, evolutionary & environmental sciences

For a reference copy of the document with all sections, see nature.com/documents/nr-reporting-summary-flat.pdf

Life sciences study design

All studies must disclose on these points even when the disclosure is negative.

Sample size	The data are from three replicates.
Data exclusions	No data were excluded.
Replication	The data are from three replicates and all attempts at replication were successful.
Randomization	Samples were tested in one group. There is no group allocation.
Blinding	Samples were tested in one group. There is no group allocation.

Reporting for specific materials, systems and methods

We require information from authors about some types of materials, experimental systems and methods used in many studies. Here, indicate whether each material, system or method listed is relevant to your study. If you are not sure if a list item applies to your research, read the appropriate section before selecting a response.

Materials & experimental systems

n/a	Involved in the study	
☒	☐	Antibodies
☒	☐	Eukaryotic cell lines
☒	☐	Palaeontology
☒	☐	Animals and other organisms
☒	☐	Human research participants
☒	☐	Clinical data

Methods

n/a	Involved in the study	
☒	☐	ChIP-seq
☐	☒	Flow cytometry
☒	☐	MRI-based neuroimaging

Flow Cytometry

Plots

Confirm that:

☐ The axis labels state the marker and fluorochrome used (e.g. CD4-FITC).

☐ The axis scales are clearly visible. Include numbers along axes only for bottom left plot of group (a 'group' is an analysis of identical markers).

☐ All plots are contour plots with outliers or pseudocolor plots.

☒ A numerical value for number of cells or percentage (with statistics) is provided.

Methodology

Sample preparation	cells were precultured overnight in LB medium and then diluted to OD600=0.2 in the same medium in the presence of corresponding inducer. The cells were then grown for 12 h and FACS sorting was performed.
Instrument	FACSAria II sorter (BD, San Jose, USA)
Software	BD FACSDiva™ software v6.1.3; FlowJo v7.6.1
Cell population abundance	A total of 350000 cells were used for sorting.
Gating strategy	The least fluorescent 80% cells (negative sorting) and most fluorescent 1% cells (positve sorting) were sorted.

☒ Tick this box to confirm that a figure exemplifying the gating strategy is provided in the Supplementary Information.
