# Supplementary material for: Developing a highly efficient hydroxytyrosol whole-cell catalyst by de-bottlenecking rate-limiting steps
Source: Nat Commun. 2020 Mar 23;11:1515. doi: 10.1038/s41467-020-14918-5 (PMC7090077; doi:10.1038/s41467-020-14918-5)
Supplement: Supplementary file 3 — Description of Additional Supplementary Files [file 41467_2020_14918_MOESM3_ESM.pdf]

Description of Additional Supplementary Files

File name: Supplementary Data 1
Description: Strains and plasmids used in this study

File name: Supplementary Data 2
Description: Primers used in this study

File name: Supplementary Data 3
Description: Sequence of the genes after codon optimization
